# Obesity, Psychological Distress, and Resting State Connectivity of the Hippocampus and Amygdala Among Women With Early-Stage Breast Cancer

**DOI:** 10.3389/fnhum.2022.848028

**Published:** 2022-04-01

**Authors:** Shannon D. Donofry, Alina Lesnovskaya, Jermon A. Drake, Hayley S. Ripperger, Alysha D. Gilmore, Patrick T. Donahue, Mary E. Crisafio, George Grove, Amanda L. Gentry, Susan M. Sereika, Catherine M. Bender, Kirk I. Erickson

**Affiliations:** ^1^Psychiatry and Behavioral Health Institute, Allegheny Health Network, Pittsburgh, PA, United States; ^2^Department of Psychology, University of Pittsburgh, Pittsburgh, PA, United States; ^3^Center for the Neural Basis of Cognition, University of Pittsburgh and Carnegie Mellon University, Pittsburgh, PA, United States; ^4^Bloomberg School of Public Health, Johns Hopkins University, Baltimore, MD, United States; ^5^Department of Health and Exercise Science, Colorado State University, Fort Collins, CO, United States; ^6^School of Nursing, University of Pittsburgh, Pittsburgh, PA, United States; ^7^Graduate School of Public Health, University of Pittsburgh, Pittsburgh, PA, United States; ^8^Clinical and Translational Science Institute, University of Pittsburgh, Pittsburgh, PA, United States

**Keywords:** amygdala, anxiety, breast cancer, depression, hippocampus, obesity, post-menopausal, resting state functional connectivity

## Abstract

**Objective:**

Overweight and obesity [body mass index (BMI) ≥ 25 kg/m^2^] are associated with poorer prognosis among women with breast cancer, and weight gain is common during treatment. Symptoms of depression and anxiety are also highly prevalent in women with breast cancer and may be exacerbated by post-diagnosis weight gain. Altered brain function may underlie psychological distress. Thus, this secondary analysis examined the relationship between BMI, psychological health, and resting state functional connectivity (rsFC) among women with breast cancer.

**Methods:**

The sample included 34 post-menopausal women newly diagnosed with Stage 0-IIa breast cancer (*M*age = 63.59 ± 5.73) who were enrolled in a 6-month randomized controlled trial of aerobic exercise vs. usual care. At baseline prior to randomization, whole-brain analyses were conducted to evaluate the relationship between BMI and seed-to-voxel rsFC of the hippocampus and amygdala. Connectivity values from significant clusters were then extracted and examined as predictors of self-reported depression and anxiety.

**Results:**

Mean BMI was in the obese range (*M* = 31.83 ± 6.62). For both seeds examined, higher BMI was associated with lower rsFC with regions of prefrontal cortex (PFC), including ventrolateral PFC (vlPFC), dorsolateral PFC, and superior frontal gyrus (*z* range = 2.85–4.26). Hippocampal connectivity with the vlPFC was negatively correlated with self-reported anxiety (β = 0.47, *p* < 0.01).

**Conclusion:**

Higher BMI was associated with lower hippocampal and amygdala connectivity to regions of PFC implicated in cognitive control and emotion regulation. BMI-related differences in hippocampal and amygdala connectivity following a recent breast cancer diagnosis may relate to future worsening of psychological functioning during treatment and remission. Additional longitudinal research exploring this hypothesis is warranted.

## Introduction

Overweight and obesity (body mass index [BMI] ≥ 25 kg/m^2^) are highly prevalent conditions affecting 71% of United States adults (Hales, 2017), and are associated with increased mortality and elevated risk for a myriad of physical (Pi-Sunyer, 2009) and psychological (Mather et al., 2009) illnesses. Excess weight is now recognized as a risk factor for several cancer subtypes (Vucenik and Stains, 2012). Recent estimates suggest that cancers associated with overweight and obesity account for 40% of all incident cancer cases diagnosed in the United States (Lauby-Secretan et al., 2016). Breast cancer is among those most robustly associated with obesity, particularly estrogen and progesterone hormone receptor-positive (HR+) breast cancers (Neuhouser et al., 2015) occurring more commonly during the post-menopausal period. Indeed, post-menopausal breast cancer risk is 20–40% higher in women who have overweight or obesity (Neuhouser et al., 2015). Moreover, obesity is associated with higher probability of metastatic disease recurrence and lower overall and cancer-related survival rates (Protani et al., 2010). Unfortunately, a sizable majority of women experience weight gain following breast cancer treatment, and return to pre-treatment weight is uncommon (Makari-Judson et al., 2014). Treatment-induced weight gain may therefore increase vulnerability for co-morbid illnesses such as cardiovascular disease and contribute to less favorable cancer-related outcomes among women with overweight and obesity. Importantly, breast cancer is the most commonly diagnosed of all cancer subtypes and up to 30% of all cancer-related deaths in women are attributable to breast cancer (Ferlay et al., 2015). Thus, both obesity and breast cancer represent significant public health challenges that jointly affect millions of women, underscoring the critical need for additional research to improve understanding of the relationship between obesity and breast cancer risk and prognosis.

Psychological distress is common in obesity and breast cancer and may exert a deleterious effect on treatment, prognosis, and quality of life. Independently of medical co-morbidities such as breast cancer, obesity increases risk for major depressive disorder (MDD) (Luppino et al., 2010), and elevated depressive symptoms have been associated with reduced likelihood of successful weight loss (Wing and Phelan, 2005). Among women with breast cancer, symptoms of depression and anxiety are highly prevalent, with data from two large meta-analyses indicating that 30–40% of women endorse such symptoms (Hashemi et al., 2020). Although psychological distress following diagnosis with breast cancer may be considered normative given the life-threatening nature of the illness, diagnosable mood and anxiety disorders are also prevalent among women with breast cancer (Fann et al., 2008; Fatiregun et al., 2016). Further, symptoms of depression and anxiety prospectively predict reduced health-related quality of life and physical functioning one year after diagnosis (Faller et al., 2017), and increased risk for cancer recurrence and all-cause mortality (Wang et al., 2020), highlighting the prognostic significance of psychological distress. However, there has been limited research exploring the mechanisms by which obesity may influence psychological functioning in women with breast cancer, impeding the development and implementation of treatments targeting psychological distress in this population.

Functional changes in brain regions such as the amygdala and hippocampus may underlie the aforementioned psychological symptoms frequently observed in breast cancer and obesity. The amygdala supports detection of behaviorally salient environmental cues, and orchestrates a cascade of processes that facilitate adaptive responding to these cues, while the hippocampus plays an instrumental role in memory encoding and associative learning (Yang and Wang, 2017). Both MDD and anxiety disorders have been associated with exaggerated reactivity of the amygdala to stimuli with ambiguous or negative emotional valence (Etkin and Wager, 2007; Groenewold et al., 2013). Structural and functional abnormalities in the hippocampus have also been documented in MDD and anxiety disorders, and are correlated with cognitive and emotional impairments characteristic of these conditions (Kaiser et al., 2015; Doré et al., 2018). In the context of obesity, functional alterations in these regions have been implicated in disruptions of self-regulation, reward valuation, self-directed thinking, and homeostatic control (Donofry et al., 2020a,b), and may also contribute to heightened vulnerability for depression and anxiety among individuals with obesity (Milaneschi et al., 2019). Thus, alterations in the functioning of the amygdala and hippocampus may mediate the development of psychological impairments among women with breast cancer, particularly those presenting with overweight and obesity. However, this hypothesis has yet to be evaluated, representing a critical scientific gap.

Neuroimaging studies in breast cancer have predominantly focused on examining neural correlates of cancer-related cognitive impairment. These studies have found that breast cancer is associated with reduced activation in regions that support executive control processes and memory, including prefrontal cortex (PFC), hippocampus, and posterior parietal cortex (López Zunini et al., 2013; Peukert et al., 2020), with some studies suggesting that heightened inflammation related to breast cancer diagnosis and treatment contributes to changes in brain health and function (Kesler et al., 2013; Peukert et al., 2020). Further, evidence suggests that breast cancer may disrupt the organization of functional brain networks when measured at rest in the absence of external input or task demands. Using functional connectivity approaches to quantify signal correlation among spatially distributed brain regions, previous research has shown that breast cancer, as well as its treatment, is associated with alterations in major resting state networks, including the default mode network (DMN) and the executive control network (ECN) (Hampson et al., 2015; Miao et al., 2016). However, despite compelling evidence that obesity is associated with a greater prevalence of negative breast cancer outcomes and may increase vulnerability for psychological distress, there have been no studies examining the association between obesity and brain network connectivity in breast cancer. Thus, it remains unclear whether the relationship between obesity and functional network organization in the context of breast cancer diagnosis and treatment are similar to or differ from patterns previously observed in individuals with obesity free of a cancer diagnosis, or whether the combined impact of obesity and breast cancer on functional network organization is related to vulnerability to psychological distress in the early stages of the disease course. To address these gaps, the present study aimed to examine the association of BMI with seed-based resting state functional connectivity (rsFC) of the hippocampus and amygdala among women recently diagnosed with breast cancer, and to determine whether BMI-related differences in resting state connectivity were related to indices of psychological health. Based on prior investigations of the association between BMI and resting connectivity (Donofry et al., 2020a), it was hypothesized that higher BMI would be associated with lower whole-brain connectivity of the hippocampus and amygdala, and that lower connectivity would be associated with higher self-reported ratings of depression and anxiety.

## Materials and Methods

### Participants

The sample included post-menopausal women recently diagnosed with Stage 0-IIIa breast cancer who were enrolled in a 6-month randomized controlled trial of aerobic exercise (ClinicalTrials.gov NCT01500356), which is still currently ongoing. To be eligible, participants had to be diagnosed with HR+ breast cancer as confirmed by their medical oncologist. Exclusion criteria included being pre-menopausal, prior treatment with cancer chemotherapy, central nervous system radiation, or intrathecal therapy, clinical evidence of distant metastases, breast cancer surgery complications (e.g., wound dehiscence), reconstructive surgery, ineligibility for aromatase inhibitor therapy, recent falls or use of an assisted walking device, comorbid medical conditions that would preclude engagement in aerobic exercise (e.g., coronary artery disease), current use of hormone replacement therapy, history of neurologic conditions (e.g., stroke), hospitalization for a psychiatric illness in the previous 2 years, substance use or eating disorder history, age-expected cardiorespiratory fitness >50th percentile, and age above 80 years. MRI-specific exclusion criteria included claustrophobia and presence of irremovable metal implants. The present investigation focused on data drawn from baseline assessments completed prior to the intervention among a subsample of women who were eligible for and consented to undergo functional MRI (fMRI) scanning (*N* = 34, 34% of total sample who completed baseline evaluation). Women who completed fMRI assessments did not differ significantly from the overall sample enrolled to date on any clinical (i.e., mood, anxiety, or cancer-related) or demographic indicator (*p*s > 0.22). Although the larger trial included women with Stage III breast cancer, the highest severity diagnosis in the neuroimaging sample was Stage IIa. Informed consent was obtained in accordance with the guidelines of the Declaration of World Medical Association (1991; p. 1194) and the institutional review boards of the institutions at which this investigation was conducted (institutional approval numbers: STUDY19080223; STUDY2016_00000197). Baseline neuroimaging assessments were conducted between November 2016 and December 2019.

### Assessments

#### Cardiorespiratory Fitness

Cardiorespiratory fitness was evaluated to determine eligibility using a sub-maximal graded treadmill exercise test as described by Gentry et al. (2018). Briefly, treadmill speed was self-selected in 0.5 mile per hour (mph) increments within a 2.0–4.0 mph range. Incline grade was increased in 1% increments every 60 s until one of three criteria were met: (1) participants reached 85% of their age-predicted maximum heart rate (220-age), (2) participants taking a beta blocker rated their perceived exertion as 15 or higher on the Borg scale (Borg, 1982), or (3) volitional exhaustion. During the test, expired air was collected *via* mouthpiece and nose clip to evaluate oxygen consumption, which was used to estimate maximal oxygen consumption (VO_2 *max*_).

#### Depressive Symptoms

Depressive symptoms were assessed using the Beck Depression Inventory-II (BDI-II) (Beck et al., 1996), a 21-item self-report measure of the severity of common depressive symptoms in the past 2 weeks rated along a 0–3 Likert scale. Responses are summed to yield a total symptom score (maximum possible score of 63) with higher scores reflecting more severe depressive symptoms. The BDI-II has demonstrated adequate reliability and validity in a number of populations, including among women with breast cancer (Love et al., 2004).

### Anxiety Symptoms

Anxiety was evaluated using the Patient-Reported Outcomes Measurement Information System (PROMIS) Emotional Distress–Anxiety Short Form (Pilkonis et al., 2011), an 8-item instrument that queries respondents on the frequency of anxiety symptoms in the past week. Symptoms are rated on a 1 (“Never”) to 5 (“Always”) Likert scale and responses are summed to obtain a raw total score, which is then converted to a standardized T-score. Higher scores are indicative of more severe symptoms of anxiety.

#### Body Mass Index

Participants’ weight and height were obtained in pounds and inches using a calibrated digital scale and wall-mounted stadiometer (Health-O-Meter 500KL Digital Scale), respectively. These values were converted to kilograms (kg) and meters (m) and used to calculate BMI according to the standard formula: kg/m^2^.

### MRI Data Acquisition and Pre-processing

MRI sequences were collected using a Siemens Verio 3T scanner (Siemens Medical Solutions USA, Inc., Malvern, PA, United States) outfitted with a 32-channel head coil. For the resting state scan, 210 *T*2*-weighted volumes were obtained for each participant using an EPI pulse sequence with blood oxygenation level-dependent (BOLD) contrast (time repetition = 1540 ms, echo time = 25 ms, flip angle = 90°, FOV = 224 mm). Thirty slices were collected at 3.5 mm thickness in the axial plane in the ventral to dorsal direction. High resolution *T*1-weighted anatomical volumes were also collected in the sagittal plane using a magnetization-prepared rapid gradient-echo (MPRAGE) sequence for each participant (176 slices, slice thickness = 1 mm, time repetition = 1900 ms, echo time = 2.93 ms, flip angle = 9°, voxel dimensions = 1 mm^3^, FOV = 250 mm). After reconstruction, data were preprocessed using FEAT version 6.00, part of FSL (FMRIB’s Software Library^1^). Motion correction was conducted using MCFLIRT (Jenkinson et al., 2002), with the middle image in the sequence designated as the reference. A bandpass temporal filter between 0.001 and 0.01 Hz was applied to remove high frequency noise attributable to physiological processes (e.g., respiration) and low frequency noise due to scanner drift. Images were spatially smoothed with a 6-mm full-width half-maximum 3-dimensional Gaussian kernel. Non-brain matter (i.e., skull) was removed using the robust brain extraction technique [BET (Smith, 2002)]. Mean functional images were registered to *T*1-weighted and MNI space (Montreal Neurological Institute–International Consortium for Brain Mapping) images applying 7- and 12-parameter affine transformations, respectively, using FMRIB’s linear image registration tool [FLIRT (Jenkinson and Smith, 2001; Jenkinson et al., 2002)]. No errors were observed in image registration.

### Resting State Functional Connectivity Analyses

A seed-based approach was used to assess whole-brain rsFC, with the hippocampus and amygdala designated as seeds in two separate analyses. Seeds were structurally defined using the Harvard–Oxford Subcortical Atlas. Using the FSL FIRST tool (Patenaude et al., 2011), automated segmentation of subcortical structures was performed to define seed masks for the hippocampus and amygdala. Masks for the left and right hemisphere of each seed were merged to create masks representing the total structure of each seed. Seed masks were then converted to native space for each individual, after which the BOLD signal time series for the seeds were extracted from each individual’s preprocessed data. The mean time course of each seed was entered into a multiple linear regression model to evaluate BOLD signal covariation between the seed region and all voxels across the brain. To reduce the influence of physiological and motion confounds on estimates of connectivity, individual-level GLMs also included the global signal, signal from white matter and cerebrospinal fluid, as well as standard and extended estimates of motion displacement (standard motion parameters with their derivatives and the square of their derivatives) as regressors of no interest. Beta values derived from these individual-level analyses were then forwarded to higher level GLMs to examine whether BMI was associated with variation in seed-to-voxel correlations at rest. Age, education, and mean framewise displacement values were included as covariates in group-level models. A cluster-defining family-wise error threshold of *p* < 0.05 were applied to all statistical parametric maps.

### Relationship Between Resting Connectivity and Psychological Functioning

To assess the relationship between estimates of functional connectivity and psychological functioning, regions of interest (ROIs) were first identified based on local maxima within clusters exhibiting significant BMI-dependent signal covariation with the seed regions. For each participant, covariation between the time series of each identified ROI and the time series of the seed region were estimated using pairwise correlation coefficients, which were then normalized using a Fisher *r*-to-*z* transformation. The FSL featquery function was used to extract the mean parameter estimates within a 10 mm sphere around local maxima coordinates. Parameter estimates were then exported to a database that included clinical and demographic data. To adjust for positive skew, both BDI-II and PROMIS Anxiety scores were transformed using a natural logarithm function to approximate a normal distribution. Descriptive analyses were conducted to examine sample characteristics. Multiple linear regression analyses were then performed to examine whether (1) BMI was associated with BDI-II or PROMIS Anxiety scores and (2) seed-to-ROI resting state connectivity values were associated with BDI-II and PROMIS Anxiety scores. Age and years of education were included as covariates in all models. To determine whether regression assumptions were met, the Global Validation of Linear Model Assumptions [gvlma (Peña and Slate, 2006)] package was used to perform regression analyses, and model fit was evaluated using the overall *F*-test. Regression diagnostic plots were also visually inspected. Presence of outliers was determined *via* Cook’s distance values using a cutoff of ≥ 0.5; no values exceeded this threshold for any analysis. For all tests, the level of statistical significance was set at *p* < 0.05 and standardized coefficients were selected for reporting significant effects. Analyses were conducted in R Studio (Racine, 2012) using R version 4.0.3 (R Core Team, 2020) and scatterplots were created using the package “ggplot2” (Wickham et al., 2016).

## Results

### Sample Characteristics

The neuroimaging subsample was comprised of post-menopausal women (*M*_*age*_ = 63.59, *SD* = 5.73) with stage 0-IIa HR+ breast cancer. Participants were predominantly White (82%) and non-Hispanic (97%). Mean BMI for the sample was in the obese range (*M* = 31.83, *SD* = 6.62), with 85% (*n* = 29) of women having a BMI of 25 or greater. Lower age (β = –0.41, *p* < 0.01) and higher framewise displacement (β = 0.71, *p* < 0.01) were associated with higher BMI. BMI was not significantly related to overall cancer stage, BDI-II scores, PROMIS Anxiety scores, race, or educational attainment (*p*s > 0.09). Participants reported mild symptoms of depression (*M* = 5.17, *SD* = 4.20, range = 0–17) and anxiety (*M* = 46.40, *SD* = 9.05, range = 37–66). Additional demographic and clinical characteristics are presented in Table 1.

**TABLE 1:** Demographic and clinical characteristics of the sample (*N* = 34).

	Mean (*SD)*
Age (years)	63.59 (5.73)
BMI (kg/m^2^)	31.83 (6.62)
Education (years)	15.94 (2.88)
BDI-II score	5.17 (4.20)
PROMIS Anxiety *T*-score	46.40 (9.05)

	***N* (%)**

**Overall cancer stage**	
Stage 0 DCIS	10 (0.29)
Stage 1	17 (0.50)
Stage 2a	7 (0.21)
**T-stage**	
Tis	10 (0.29)
T1a	2 (0.06)
T1b	8 (0.24)
T1d	9 (0.27)
T2	5 (0.15)
**Race**	
White	28 (0.82)
Black or African American	4 (0.12)
Multi-racial	2 (0.15)
Hispanic ethnicity	1 (0.03)

*Percentages may not add to 100 due to rounding. BMI, body mass index; BDI-II, Beck Depression Inventory-II; PROMIS, Patient-Reported Outcomes Measurement Information System; DCIS, ductal carcinoma in situ.*

### Relationship Between Body Mass Index and Whole-Brain Resting State Connectivity

**FIGURE 1:**
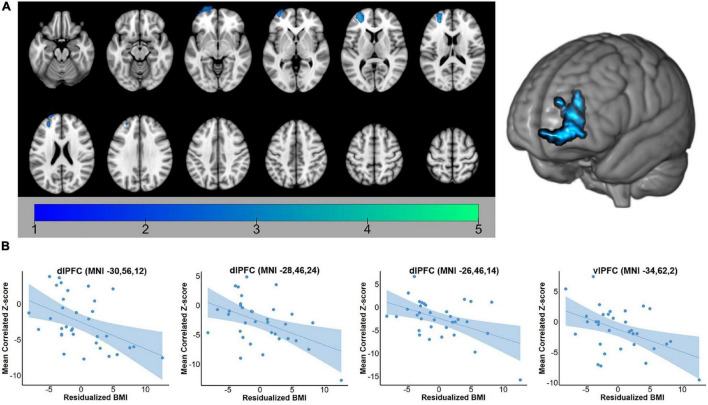
**(A)** Whole-brain map of regions exhibiting negative associations between BMI and intrinsic connectivity with the hippocampus. Map was cluster thresholded at *z* > 2.3 and *p* < 0.05. **(B)** Scatterplots of the relationship between BMI and hippocampus-to-ROI signal covariation at rest. Note that mean framewise displacement, age, and education were regressed out of BMI values prior to plotting. BMI, body mass index; dlPFC, dorsolateral prefrontal cortex; vlPFC, ventrolateral prefrontal cortex.

**FIGURE 2:**
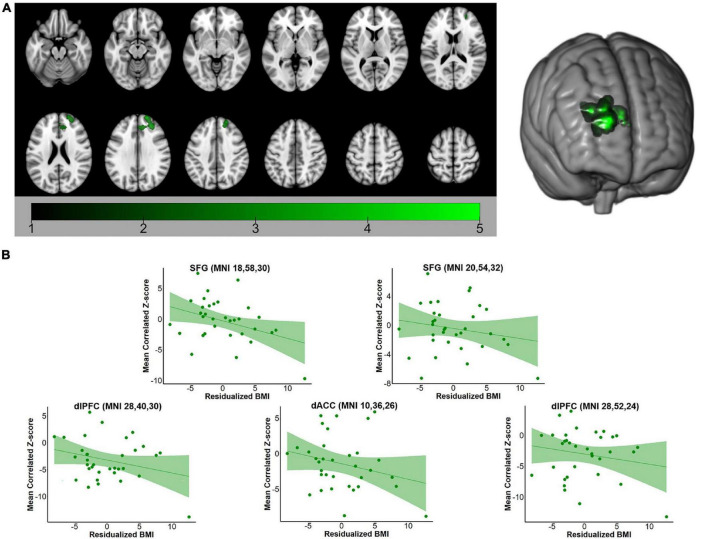
**(A)** Whole-brain map of regions exhibiting negative associations between BMI and intrinsic connectivity with the amygdala. Map was cluster thresholded at *z* > 2.3 and *p* < 0.05. **(B)** Scatterplots of the relationship between BMI and amygdala-to-ROI signal covariation at rest. Note that mean framewise displacement, age, and education were regressed out of BMI values prior to plotting. BMI, body mass index; SFG, superior frontal gyrus; dlPFC, dorsolateral prefrontal cortex; dACC, dorsal anterior cingulate cortex.

For both the hippocampus and amygdala seeds, BMI was negatively correlated with whole-brain resting state connectivity. For the hippocampal seed, higher body mass was associated with lower connectivity to a single cluster located in the left PFC (voxel-wise *z*-max = 4.26, *p* = 0.017, cluster extent = 753 voxels). As depicted in Figure 1, this cluster encompassed dorsolateral and ventrolateral PFC (vlPFC). Signal within the ROI spheres located around the peak voxels of the cluster illustrated a linear decrease in hippocampal connectivity as BMI increased. For the amygdala seed, BMI was inversely associated with resting connectivity of a single cluster of regions located in the right PFC (voxel-wise *z*-max = 3.90, *p* = 0.011, cluster extent = 773 voxels) that included dlPFC, superior frontal gyrus (SFG), and dorsal anterior cingulate cortex (dACC). Similar to the patterns observed with the hippocampal seed, signal covariation between the amygdala and the ROI spheres located around the peak voxels of the cluster illustrated a linear decrease in hippocampal connectivity as BMI increased (Figure 2). Further details regarding the regions that exhibited significant BMI-related associations with resting connectivity with each seed are described in Table 2.

**TABLE 2:** MNI coordinates (in mm) of local maxima in regions of interest for which BMI was negatively correlated with intrinsic functional connectivity with each seed at rest.

Seed	Cluster extent (*p-value*)	Region of interest	Hemisphere	Local maxima *z*-score	*x*	*y*	*z*
Hippocampus	773 (0.01)						
		Dorsolateral PFC	Left	4.26	−30	56	12
		Dorsolateral PFC	Left	3.68	−26	46	14
		Dorsolateral PFC	Left	3.54	−28	46	24
		Ventrolateral PFC	Left	3.10	−34	62	2
Amygdala	753 (0.02)						
		Superior Frontal Gyrus	Right	3.9	18	58	30
		Superior Frontal Gyrus	Right	3.63	20	54	32
		Dorsolateral PFC	Right	3.48	28	40	30
		Dorsal ACC	Right	3.23	10	36	26
		Dorsolateral PFC	Right	2.92	28	52	24

*Regions met a cluster-defining threshold of z > 2.3 and p < 0.05. Cluster extent is in voxels. The first coordinate listed under each cluster describes the location of the highest local maximum within the cluster. Additional regions with local maxima within the same cluster are listed following the strongest local maximum for that cluster. MNI, Montreal Neurological Institute; BMI, body mass index; PFC, prefrontal cortex; ACC, anterior cingulate cortex.*

### Relationship Between Seed-to-Regions of Interest Connectivity and Ratings of Mood and Anxiety

As indicated in the Methods, 10 mm spheres were created around statistical peaks in the clusters described above in order to conduct a secondary analysis examining whether BMI-related variation in functional connectivity was related to mood or anxiety. Contrary to study hypotheses, no indices of seed-to-ROI connectivity were associated with BDI-II scores for either the hippocampus or amygdala seed (hippocampus: β range = −0.12–0.21, *p*-value range = 0.25–0.93; amygdala: β range = –0.23–0.22, *p*-value range = 0.23–0.90). Regarding anxiety symptoms, hippocampal connectivity to the left vlPFC ROI was significantly related to PROMIS Anxiety scores, with lower connectivity values being associated with higher ratings of anxiety symptom severity (β = –0.47, *p* < 0.01). Hippocampal connectivity with other ROIs was unrelated to PROMIS Anxiety scores (β range = –0.25–0.23, *p*-value range = 0.17–0.24), and no index of amygdala connectivity was associated with anxiety scores (β range = –0.28–0.26, *p*-value range = 0.13–0.35). Because of the high correlation between BMI and framewise displacement, all models in which we examined the relationship between seed-to-ROI connectivity and ratings of mood and anxiety were re-analyzed with framewise displacement as an additional covariate. Inclusion of framewise displacement as a covariate in these models did not alter any results.

## Discussion

To our knowledge, the present study is the first to examine the association of overweight and obesity with rsFC among women recently diagnosed with breast cancer, and to link indices of resting connectivity to psychological functioning. Results indicated that BMI was inversely associated with hippocampal and amygdala connectivity with regions of the PFC that support executive functions such as cognitive control, emotion regulation, and goal-concordant decision making. Specifically, greater hippocampal engagement was associated with lower engagement of regions comprising the ECN, including left dorsolateral prefrontal cortex (dlPFC) and vlPFC, among women with overweight and obesity. A similar pattern was observed with the amygdala whereby higher BMI was related to weaker connectivity between the amygdala and regions of the ECN such as the right dlPFC and SFG, as well as a major node of the salience network (SN), the dACC. These findings suggest that obesity may be associated with the functional organization of major resting state networks, with such associations potentially being linked to obesity-related relationships in physical and psychological functioning among women with breast cancer; however, these findings should be considered preliminary and replicated in a large, prospective longitudinal study.

### Clinical Implications

The observation that intrinsic hippocampal connectivity to the PFC is negatively correlated with BMI is consistent with prior investigations among individuals with obesity. The hippocampus is a component of the DMN, a functional network that has been implicated in internally guided cognitive processes such as self-directed thinking, episodic memory, and social reasoning (Raichle, 2015). We have demonstrated that higher BMI is associated with weaker connectivity between the hippocampus and other regions of the DMN among midlife adults (Donofry et al., 2020a), a pattern noted in several other investigations of resting state connectivity in obesity (Doucet et al., 2018; Ding et al., 2020). Results from the present study extend these findings by suggesting that obesity may be associated with altered communication between nodes of the DMN and ECN, which may in turn relate to psychological functioning among women with breast cancer. Interactions between the hippocampus and dlPFC are thought to support several executive processes disrupted in obesity and in breast cancer, including working memory (Bähner et al., 2015), representation of event salience and relevant contextual factors (Jafarpour et al., 2019), and inhibitory control over memory retrieval (Anderson et al., 2016). Thus, associations between higher BMI and decreases in hippocampal connectivity with brain regions located in the ECN may underlie psychological distress among women recently diagnosed with breast cancer. Nevertheless, additional longitudinal research in larger samples is needed to evaluate this hypothesis.

Hippocampal disruptions have also been noted among women with breast cancer independently of weight gain or obesity, primarily in the context of cancer-related cognitive impairment. Of particular relevance is a recent study demonstrating that reduced dlPFC-hippocampal connectivity at rest was associated with poorer performance on a working memory task among pre-menopausal women undergoing hormone therapy for HR+ breast cancer (Chen et al., 2017). Prospective longitudinal studies have also demonstrated that chemotherapy treatment predicts changes in hippocampal connectivity at rest (Feng et al., 2020), with these changes in connectivity being associated with treatment-related decrements in cognitive (Feng et al., 2020) and psychological functioning (Feng et al., 2020). These findings suggest that metrics of hippocampal functional connectivity may have prognostic value among women with breast cancer. However, additional research is needed to determine how overweight and obesity prospectively influence the relationship between hippocampal connectivity and psychological outcomes over the course of cancer treatment and remission.

The present study also demonstrated that higher body mass index was associated with lower amygdala connectivity to nodes of the ECN and SN. The amygdala is part of the neurocircuitry responsible for mounting cardiovascular and neuroendocrine responses to stressful experiences, and repeated engagement of the circuitry due to chronic stressor exposure is hypothesized to contribute to the development of chronic disease (Gianaros and Jennings, 2018). Obesity and breast cancer also provoke pro-inflammatory states (Deng et al., 2016), which in turn may disrupt signaling in the amygdala. Indeed, two recent studies found that elevated concentrations of circulating inflammatory markers such as C-reactive protein are associated with higher amygdala reactivity to social threat among women who have undergone treatment for early-stage breast cancer (Muscatell et al., 2016; Leschak et al., 2020). Intriguingly, emerging evidence suggests that integrated behavioral interventions simultaneously targeting obesity and depression may modify amygdala activation and connectivity, with changes in amygdala function being related to improvements in weight and health behavior engagement (Lv et al., 2021). Together, these studies suggest that altered amygdala functioning may be mechanistically linked to obesity and breast cancer. However, as previously mentioned, these results should be considered preliminary, and additional longitudinal and intervention research is necessary to further elucidate these relationships.

### Study Limitations

There are several strengths of the present study. To our knowledge, this is the first investigation of the association of obesity with functional brain network organization among women with breast cancer, and to link obesity-related differences in functional network organization to indices of psychological functioning. Thus, the present study represents an initial step toward advancing scientific understanding of the mechanisms through which obesity may contribute to poor physical and mental health among women with breast cancer. Nevertheless, results should be interpreted in the context of several important limitations. First, the sample size was relatively small, which restricted our statistical power. Our findings should be considered preliminary until replicated in a much larger sample. Relatedly, our sample was racially homogenous and therefore not representative. Racial and ethnic minority groups are disproportionately burdened by cancer, receive inadequate care, and have higher rates of cancer recurrence and mortality (O’Keefe et al., 2015). There are also racial disparities in the assessment and management of psychiatric symptoms (Alegría et al., 2008). Thus, it is imperative that future studies be conducted in more diverse samples and aim to further understand and eliminate racial disparities in cancer treatment and outcomes. Second, because the present study did not include a control group of women free of breast cancer, nor a group with another type of cancer, we cannot resolve whether the correlation between obesity and connectivity patterns is of greater magnitude in women with breast cancer compared to those without, or those with another type of cancer. Third, the cross-sectional observational design of the study precludes inferences of causality. Prospective study designs are needed to establish the temporal relationship between obesity and functional connectivity in women with breast cancer. Indeed, it is possible that the differences in resting functional connectivity described here precede the onset of obesity instead of resulting from obesity. Only longitudinal and interventional studies are capable of testing these possible directions. Fortunately, because these data were collected in the context of a clinical trial of aerobic exercise, we will have the unique opportunity to examine whether obesity-related variations in resting state networks at baseline predicts intervention outcomes, including intervention and treatment-related changes in psychological functioning. These follow-up analyses will aid in clarifying whether patterns of resting connectivity observed at the time of breast cancer diagnosis predict clinically meaningful outcomes. Fourth, although BMI is predictive of a number of disease outcomes (Manson et al., 1990; Calle et al., 2003), is moderately correlated with objective measures of adiposity (Romero-Corral et al., 2008), and is easily and affordably measured, it would have been preferable to have a direct measure of central adiposity. Fifth, given the focus of this study on exploring the impact of obesity on resting state connectivity in women with breast cancer, the ROIs used in the correlation with mood and anxiety were defined based on the significant relationship with BMI. This may have precluded the identification of patterns of brain connectivity unrelated to BMI but potentially associated with psychological distress in this sample. Finally, our sample was limited to post-menopausal women with breast cancer who were eligible for aromatase inhibitor therapy. Future studies should consider examining the relationship between overweight and obesity and brain network organization among younger women with breast cancer as well in other cancers linked with obesity that affect both men and women, including colorectal and lung cancers.

## Summary and Conclusion

To conclude, our results indicate that higher BMI is associated with weaker resting connectivity between subcortical regions involved in emotion processing and regions of prefrontal cortex that support executive functions such as cognitive and emotion regulation. Further, hippocampal connectivity with the vlPFC was inversely associated with anxiety symptom severity, suggesting that obesity-related disruption of hippocampal connectivity with the prefrontal cortex may contribute to the emergence of psychological distress following diagnosis with breast cancer. Additional research is necessary to determine whether BMI-related differences in hippocampal and amygdala connectivity following a recent breast cancer diagnosis relate to future worsening of psychological functioning during treatment and remission. It will also be important to examine whether altered functional network organization at the time of diagnosis predicts disease course, particularly of negative outcomes that have been linked to overweight and obesity. Continued research in this area has the potential to elucidate the mechanisms through which obesity influences health and wellbeing among women diagnosed with breast cancer, which may contribute to the development of novel treatment and prevention strategies targeting weight-related disparities in cancer morbidity and mortality.

## Data Availability Statement

The raw data supporting the conclusions of this article will be made available by the authors, without undue reservation.

## Ethics Statement

The studies involving human participants were reviewed and approved by University of Pittsburgh Institutional Review Board and Carnegie Mellon University Institutional Review Board. The patients/participants provided their written informed consent to participate in this study.

## Author Contributions

CB, KE, and SD: conception and study design. CB, KE, ALG, PD, MC, and GG: data collection and acquisition. SD, AL, JD, HR, and ADG: statistical analysis. SD, AL, JD, and HR: interpretation of results. SD: drafting the manuscript work. SD, KE, CB, SS, MC, PD, GG, ALG, JD, AL, and HR: revising the manuscript critically for important intellectual content. All authors approved the final version to be published and agreed to be accountable for the integrity and accuracy of all aspects of the work.

## Conflict of Interest

The authors declare that the research was conducted in the absence of any commercial or financial relationships that could be construed as a potential conflict of interest.

## Publisher’s Note

All claims expressed in this article are solely those of the authors and do not necessarily represent those of their affiliated organizations, or those of the publisher, the editors and the reviewers. Any product that may be evaluated in this article, or claim that may be made by its manufacturer, is not guaranteed or endorsed by the publisher.

## Footnotes

1
http://www.fmrib.ox.ac.uk/fsl/

## References

[B1] Alegría M. Chatterji P. Wells K. Cao Z. Chen C. Takeuchi D. (2008). Disparity in depression treatment among racial and ethnic minority populations in the United States. *Psychiatr. Serv.* 59 1264–1272. 10.1176/ps.2008.59.11.1264 18971402 PMC2668139

[B2] Anderson M. C. Bunce J. G. Barbas H. (2016). Prefrontal–hippocampal pathways underlying inhibitory control over memory. *Neurobiol. Learn. Mem.* 134 145–161. 10.1016/j.nlm.2015.11.008 26642918 PMC5106245

[B3] Bähner F. Demanuele C. Schweiger J. Gerchen M. F. Zamoscik V. Ueltzhöffer K. (2015). Hippocampal–Dorsolateral prefrontal coupling as a species-conserved cognitive mechanism: a human translational imaging study. *Neuropsychopharmacology* 40 1674–1681. 10.1038/npp.2015.13 25578799 PMC4915249

[B4] Beck A. T. Steer R. A. Brown G. K. (1996). *Beck Depression Inventory–II.* San Antonio, TX: Psychological Corporation.

[B5] Borg G. A. V. (1982). Psychophysical bases of perceived exertion. *Med. Sci. Sports Exerc.* 14 377–381. 10.1249/00005768-198205000-00012 7154893

[B6] Calle E. E. Rodriguez C. Walker-Thurmond K. Thun M. J. (2003). Overweight, obesity, and mortality from cancer in a prospectively studied Cohort of U.S. Adults. *N. Engl. J. Med.* 348 1625–1638. 10.1056/NEJMoa021423 12711737

[B7] Chen X. He X. Tao L. Li J. Wu J. Zhu C. (2017). The Working memory and dorsolateral prefrontal-hippocampal functional connectivity changes in long-term survival breast cancer patients treated with Tamoxifen. *Int. J. Neuropsychopharmacol.* 20 374–382. 10.1093/ijnp/pyx008 28177081 PMC5417059

[B8] Deng T. Lyon C. J. Bergin S. Caligiuri M. A. Hsueh W. A. (2016). Obesity, Inflammation, and Cancer. *Annu. Rev. Pathol. Mech. Dis.* 11 421–449. 10.1146/annurev-pathol-012615-044359 27193454

[B9] Ding Y. Ji G. Li G. Zhang W. Hu Y. Liu L. (2020). Altered interactions among resting-state networks in individuals with obesity. *Obesity* 28 601–608. 10.1002/oby.22731 32090510 PMC7098432

[B10] Donofry S. D. Jakicic J. M. Rogers R. J. Watt J. C. Roecklein K. A. Erickson K. I. (2020a). Comparison of food cue-evoked and resting-state functional connectivity in obesity. *Psychosom. Med.* 82 261–271. 10.1097/PSY.0000000000000769 32267660 PMC8057093

[B11] Donofry S. D. Stillman C. M. Erickson K. I. (2020b). A review of the relationship between eating behavior, obesity and functional brain network organization. *Soc. Cogn. Affect. Neurosci.* 15 1157–1181. 10.1093/scan/nsz085 31680149 PMC7657447

[B12] Doré B. P. Rodrik O. Boccagno C. Hubbard A. Weber J. Stanley B. (2018). Negative autobiographical memory in depression reflects elevated amygdala-hippocampal reactivity and Hippocampally associated emotion regulation. *Biol. Psychiatry Cogn. Neurosci. Neuroimaging* 3 358–366. 10.1016/j.bpsc.2018.01.002 29628068

[B13] Doucet G. E. Rasgon N. McEwen B. S. Micali N. Frangou S. (2018). Elevated Body Mass Index is Associated with Increased Integration and Reduced Cohesion of Sensory-Driven and Internally Guided Resting-State Functional Brain Networks. *Cereb. Cortex* 28 988–997. 10.1093/cercor/bhx008 28119342 PMC6059200

[B14] Etkin A. Wager T. D. (2007). Functional neuroimaging of anxiety: a meta-analysis of emotional processing in PTSD, Social Anxiety Disorder, and Specific Phobia. *Am. J. Psychiatry* 164 1476–1488. 10.1176/appi.ajp.2007.07030504 17898336 PMC3318959

[B15] Faller H. Strahl A. Richard M. Niehues C. Meng K. (2017). Symptoms of depression and anxiety as predictors of physical functioning in breast cancer patients. A prospective study using path analysis. *Acta Oncol.* 56 1677–1681. 10.1080/0284186X.2017.1333630 28595474

[B16] Fann J. R. Thomas-Rich A. M. Katon W. J. Cowley D. Pepping M. McGregor B. A. (2008). Major depression after breast cancer: a review of epidemiology and treatment. *Gen. Hosp. Psychiatry* 30 112–126. 10.1016/j.genhosppsych.2007.10.008 18291293

[B17] Fatiregun O. A. Olagunju A. T. Erinfolami A. R. Fatiregun O. A. Arogunmati O. A. Adeyemi J. D. (2016). Anxiety disorders in breast cancer: prevalence, types, and determinants. *J. Psychosoc. Oncol.* 34 432–447. 10.1080/07347332.2016.1196805 27269867

[B18] Feng Y. Tuluhong D. Zhao S. Zheng L. J. Chen T. Lu G. M. (2020). Postchemotherapy hippocampal functional connectivity patterns in patients with breast cancer: a longitudinal resting state functional MR imaging study. *Brain Imaging Behav.* 14 1456–1467. 10.1007/s11682-019-00067-x 30877468

[B19] Ferlay J. Soerjomataram I. Dikshit R. Eser S. Mathers C. Rebelo M. (2015). Cancer incidence and mortality worldwide: sources, methods and major patterns in GLOBOCAN 2012. *Int. J. Cancer* 136 E359–E386. 10.1002/ijc.29210 25220842

[B20] Gentry A. L. Erickson K. I. Sereika S. M. Casillo F. E. Crisafio M. E. Donahue P. T. (2018). Protocol for Exercise Program in Cancer and Cognition (EPICC): a randomized controlled trial of the effects of aerobic exercise on cognitive function in postmenopausal women with breast cancer receiving aromatase inhibitor therapy. *Contemp. Clin. Trials* 67 109–115. 10.1016/j.cct.2018.02.012 29501739 PMC5877817

[B21] Gianaros P. J. Jennings J. R. (2018). Host in the Machine: a neurobiological perspective on psychological stress and cardiovascular disease. *Am. Psychol.* 73 1031–1044. 10.1037/amp0000232 30394781 PMC6220680

[B22] Groenewold N. A. Opmeer E. M. de Jonge P. Aleman A. Costafreda S. G. (2013). Emotional valence modulates brain functional abnormalities in depression: evidence from a meta-analysis of fMRI studies. *Neurosci. Biobehav. Rev.* 37 152–163. 10.1016/j.neubiorev.2012.11.015 23206667

[B23] Hales C. M. (2017). Prevalence of obesity among adults and youth: United States, 2015–2016. *CHS Data Brief* 288 1–8. 29155689

[B24] Hampson J. P. Zick S. M. Khabir T. Wright B. D. Harris R. E. (2015). Altered resting brain connectivity in persistent cancer related fatigue. *Neuroimage Clin.* 8 305–313. 10.1016/j.nicl.2015.04.022 26106555 PMC4474178

[B25] Hashemi S.-M. Rafiemanesh H. Aghamohammadi T. Badakhsh M. Amirshahi M. Sari M. (2020). Prevalence of anxiety among breast cancer patients: a systematic review and meta-analysis. *Breast Cancer* 27 166–178. 10.1007/s12282-019-01031-9 31828585

[B26] Jafarpour A. Griffin S. Lin J. J. Knight R. T. (2019). Medial orbitofrontal cortex, dorsolateral prefrontal cortex, and hippocampus differentially represent the event saliency. *J. Cogn. Neurosci.* 31 874–884. 10.1162/jocn_a_01392 30883290 PMC6941931

[B27] Jenkinson M. Bannister P. Brady M. Smith S. (2002). Improved optimization for the robust and accurate linear registration and motion correction of brain images. *Neuroimage* 17 825–841. 10.1006/nimg.2002.1132 12377157

[B28] Jenkinson M. Smith S. (2001). A global optimisation method for robust affine registration of brain images. *Med. Image Anal.* 5 143–156. 10.1016/s1361-8415(01)00036-6 11516708

[B29] Kaiser R. H. Andrews-Hanna J. R. Wager T. D. Pizzagalli D. A. (2015). Large-scale network dysfunction in major depressive disorder: a meta-analysis of resting-state functional connectivity. *JAMA Psychiatry* 72 603–611. 10.1001/jamapsychiatry.2015.0071 25785575 PMC4456260

[B30] Kesler S. R. Wefel J. S. Hosseini S. M. H. Cheung M. Watson C. L. Hoeft F. (2013). Default mode network connectivity distinguishes chemotherapy-treated breast cancer survivors from controls. *Proc. Nat. Acad. Sci.* 110, 11600–11605. 10.1073/pnas.1214551110 23798392 PMC3710809

[B31] Lauby-Secretan B. Scoccianti C. Loomis D. Grosse Y. Bianchini F. Straif K. (2016). Body Fatness and Cancer—Viewpoint of the IARC Working Group. *N. Engl. J. Med.* 375 794–798. 10.1056/NEJMsr1606602 27557308 PMC6754861

[B32] Leschak C. J. Dutcher J. M. Haltom K. E. B. Breen E. C. Bower J. E. Eisenberger N. I. (2020). Associations between amygdala reactivity to social threat, perceived stress and C-reactive protein in breast cancer survivors. *Soc. Cogn. Affect. Neurosci.* 15 1056–1063. 10.1093/scan/nsz103 32039441 PMC7657448

[B33] López Zunini R. A. Scherling C. Wallis N. Collins B. MacKenzie J. Bielajew C. (2013). Differences in verbal memory retrieval in breast cancer chemotherapy patients compared to healthy controls: a prospective fMRI study. *Brain Imaging Behav.* 7 460–477. 10.1007/s11682-012-9213-0 23242968

[B34] Love A. W. Grabsch B. Clarke D. M. Bloch S. Kissane D. W. (2004). Screening for depression in women with metastatic breast cancer: a comparison of the beck depression inventory short form and the hospital anxiety and depression scale. *Aust. N. Z. J. Psychiatry* 38 526–531. 10.1080/j.1440-1614.2004.01385.x 15255825

[B35] Luppino F. S. de Wit L. M. Bouvy P. F. Stijnen T. Cuijpers P. Penninx B. W. J. H. (2010). Overweight, obesity, and depression: a systematic review and meta-analysis of longitudinal studies. *JAMA Psychiatry* 67 220–229. 10.1001/archgenpsychiatry.2010.2 20194822

[B36] Lv N. Lefferts W. K. Xiao L. Goldstein-Piekarski A. N. Wielgosz J. Lavori P. W. (2021). Problem-solving therapy-induced amygdala engagement mediates lifestyle behavior change in obesity with comorbid depression: a randomized proof-of-mechanism trial. *Am. J. Clin. Nutr.* 114 2060–2073. 10.21203/rs.3.rs-53210/v1 PMC8634561 34476464

[B37] Makari-Judson G. Braun B. Jerry D. J. Mertens W. C. (2014). Weight gain following breast cancer diagnosis: implication and proposed mechanisms. *World J. Clin. Oncol.* 5 272–282. 10.5306/wjco.v5.i3.272 25114844 PMC4127600

[B38] Manson J. E. Colditz G. A. Stampfer M. J. Willett W. C. Rosner B. Monson R. R. (1990). A prospective study of obesity and risk of coronary heart disease in women. *N. Engl. J. Med.* 322 882–889. 10.1056/NEJM199003293221303 2314422

[B39] Mather A. A. Cox B. J. Enns M. W. Sareen J. (2009). Associations of obesity with psychiatric disorders and suicidal behaviors in a nationally representative sample. *J. Psychosom. Res.* 66 277–285. 10.1016/j.jpsychores.2008.09.008 19302884

[B40] Miao H. Chen X. Yan Y. He X. Hu S. Kong J. (2016). Functional connectivity change of brain default mode network in breast cancer patients after chemotherapy. *Neuroradiology* 58 921–928. 10.1007/s00234-016-1708-8 27278455

[B41] Milaneschi Y. Simmons W. K. van Rossum E. F. C. Penninx B. W. (2019). Depression and obesity: evidence of shared biological mechanisms. *Mol. Psychiatry* 24 18–33. 10.1038/s41380-018-0017-5 29453413

[B42] Muscatell K. A. Eisenberger N. I. Dutcher J. M. Cole S. W. Bower J. E. (2016). Links between inflammation, amygdala reactivity, and social support in breast cancer survivors. *Brain Behav. Immun.* 53 34–38. 10.1016/j.bbi.2015.09.008 26384778 PMC5784760

[B43] Neuhouser M. L. Aragaki A. K. Prentice R. L. Manson J. E. Chlebowski R. Carty C. L. (2015). Overweight, obesity, and postmenopausal invasive breast cancer risk: a secondary analysis of the women’s health initiative randomized clinical trials. *JAMA Oncol.* 1 611–621. 10.1001/jamaoncol.2015.1546 26182172 PMC5070941

[B44] O’Keefe E. B. Meltzer J. P. Bethea T. N. (2015). Health disparities and cancer: racial disparities in cancer mortality in the United States, 2000–2010. *Front. Public Health* 3:51. 10.3389/fpubh.2015.00051 25932459 PMC4398881

[B45] Patenaude B. Smith S. M. Kennedy D. N. Jenkinson M. (2011). A Bayesian model of shape and appearance for subcortical brain segmentation. *Neuroimage* 56 907–922. 10.1016/j.neuroimage.2011.02.046 21352927 PMC3417233

[B46] Peña E. A. Slate E. H. (2006). Global validation of linear model assumptions. *J. Am. Stat. Assoc.* 101 341–354. 10.1198/016214505000000637 20157621 PMC2820257

[B47] Peukert X. Steindorf K. Schagen S. B. Runz A. Meyer P. Zimmer P. (2020). Hippocampus—related cognitive and affective impairments in patients with breast cancer—A systematic review. *Front. Oncol.* 10:147. 10.3389/fonc.2020.00147 32154164 PMC7046686

[B48] Pilkonis P. A. Choi S. W. Reise S. P. Stover A. M. Riley W. T. Cella D. (2011). Item banks for measuring emotional distress from the Patient-Reported Outcomes Measurement Information System (PROMIS^®^): depression, anxiety, and anger. *Assessment* 18 263–283. 10.1177/1073191111411667 21697139 PMC3153635

[B49] Pi-Sunyer X. (2009). The medical risks of obesity. *Postgrad. Med.* 121 21–33. 10.3810/pgm.2009.11.2074 19940414 PMC2879283

[B50] Protani M. Coory M. Martin J. H. (2010). Effect of obesity on survival of women with breast cancer: systematic review and meta-analysis. *Breast Cancer Res. Treat.* 123 627–635. 10.1007/s10549-010-0990-0 20571870

[B51] R Core Team (2020). *R: A Language and Environment for Statistical Computing.* Vienna: R Foundation for Statistical Computing.

[B52] Racine J. S. (2012). RStudio: a platform-independent IDE for R and Sweave. *J. Appl. Econom.* 27 167–172. 10.1002/jae.1278

[B53] Raichle M. E. (2015). The brain’s default mode network. *Annu. Rev. Neurosci.* 38 433–447. 10.1146/annurev-neuro-071013-014030 25938726

[B54] Romero-Corral A. Somers V. K. Sierra-Johnson J. Thomas R. J. Collazo-Clavell M. L. Korinek J. (2008). Accuracy of body mass index in diagnosing obesity in the adult general population. *Int. J. Obes.* 32 959–966. 10.1038/ijo.2008.11 18283284 PMC2877506

[B55] Smith S. M. (2002). Fast robust automated brain extraction. *Hum. Brain Mapp.* 17 143–155. 10.1002/hbm.10062 12391568 PMC6871816

[B56] Vucenik I. Stains J. P. (2012). Obesity and cancer risk: evidence, mechanisms, and recommendations. *Ann. N. Y. Acad. Sci.* 1271 37–43. 10.1111/j.1749-6632.2012.06750.x 23050962 PMC3476838

[B57] Wang X. Wang N. Zhong L. Wang S. Zheng Y. Yang B. (2020). Prognostic value of depression and anxiety on breast cancer recurrence and mortality: a systematic review and meta-analysis of 282,203 patients. *Mol. Psychiatry* 25 3186–3197. 10.1038/s41380-020-00865-6 32820237 PMC7714689

[B58] Wickham H. Chang W. Henry L. Pedersen T. Takahashi K. Wilke C. (2016). *ggplot2: Elegant Graphics for Data Analysis.* New York, NY: Springer-Verlag.

[B59] Wing R. R. Phelan S. (2005). Long-term weight loss maintenance. *Am. J. Clin. Nutr.* 82 222S–225S. 10.1093/ajcn/82.1.222S 16002825

[B60] World Medical Association (1991). Declaration of Helsinki. *Law Med. Health Care* 19, 264–265. 11642954

[B61] Yang Y. Wang J.-Z. (2017). From structure to behavior in basolateral amygdala-hippocampus circuits. *Front. Neural Circuits* 11:86. 10.3389/fncir.2017.00086 29163066 PMC5671506

